# Feasibility of the Archercise biofeedback device to strengthen foot musculature

**DOI:** 10.1186/s13047-020-00394-z

**Published:** 2020-07-13

**Authors:** Penelope J. Latey, John Eisenhuth, Marnee J. McKay, Claire E. Hiller, Premala Sureshkumar, Elizabeth J. Nightingale, Joshua Burns

**Affiliations:** 1 The University of Sydney, School of Health Sciences, Faculty of Medicine and Health, Lidcombe, New South Wales 2141 Australia; 2 The University of Sydney, Concord Clinical School, Concord, New South Wales 2139 Australia; 3 Children’s Hospital at Westmead, New South Wales, 2145 Australia

**Keywords:** Intrinsic foot muscles, Toe flexion, Exercise adherence, Motor skills, Biofeedback

## Abstract

**Background:**

Foot muscle weakness can produce foot deformity, pain and disability. Toe flexor and foot arch exercises focused on intrinsic foot muscle strength and functional control may mitigate the progression of foot deformity and disability. Ensuring correct exercise technique is challenging due to the specificity of muscle activation required to complete some foot exercises. Biofeedback has been used to improve adherence, muscle activity and movement patterns. We investigated the feasibility of using a novel medical device, known as “Archercise”, to provide real-time biofeedback of correct arch movement via pressure change in an inflatable bladder, and foot location adherence via sensors embedded in a footplate during four-foot exercises.

**Methods:**

Thirty adults (63% female, aged 23–68 years) performed four-foot exercises twice on the Archercise sensor footplate alone and then with biofeedback. One-way repeated measures ANOVA with pairwise comparisons were computed to assess the consistency of the exercise protocol between trial 1 and trial 2 (prior to biofeedback), and the effectiveness of the Archercise biofeedback device between trial 2 and trial 3 (with biofeedback). Outcome measures were: Arch movement exercises of arch elevation and lowering speed, controlled arch elevation, controlled arch lowering, endurance of arch elevation; Foot location adherence was determined by percentage of time the great toe, fifth toe and heel contacted footplate sensors during testing and were analysed with paired sample t-tests. Participant survey comments on the use of Archercise with biofeedback were reported thematically.

**Results:**

Seventeen (89%) arch movement and foot location variables were collected consistently with Archercise during the foot exercises. Archercise with biofeedback improved foot location adherence for all exercises (*p* = 0.003–0.008), coefficient of determination for controlled arch elevation (*p* < 0.0001) and endurance area ratio (*p* = 0.001). Twenty-nine (97%) participants reported Archercise with biofeedback, helped correct exercise performance.

**Conclusions:**

Archercise is a feasible biofeedback device to assist healthy participants without foot pathologies perform foot doming exercises.

**Trial registration:**

Australian New Zealand Clinical Trials Registry (ANZCTR): 12616001559404. Registered 11 November 2016, http://www.ANZCTR.org.au/ACTRN12616001559404p.aspx

## Background

Foot muscle weakness resulting from disease, inactivity and aging [1], can produce foot deformity, pain and disability [2–4]. For example, peripheral neuropathy causes foot muscle weakness, reduced foot muscle volume and disabling cavovarus foot deformity [5]. Several studies have also reported an association between loss of toe flexor strength or intrinsic foot muscle size, and high intensity foot pain [3], plantar fasciitis [6, 7] and painful hallux valgus [8, 9].

Toe flexor and foot arch exercises focused on maintaining intrinsic foot muscle strength and functional control [10] may mitigate the progression of foot deformity such as hallux valgus [11]. Slowing the development of disabling and painful deformities by addressing foot muscle weakness may limit functional impairments and improve quality of life [8, 12, 13]. Strengthening foot musculature may also reduce the associated risk of falls and loss of balance in older people [4, 14]. Supervised practice of the short foot exercise in particular, which is performed by approximating the metatarsal heads towards the heels without toe flexion, improves static unilateral balance [15] and increases cross-sectional area of abductor hallucis in people with pes planus [16]. Even though toe flexor and foot arch exercises are routinely prescribed to improve foot muscle strength, maintaining adherence to exercise is challenging [17] thereby limiting it impact on health outcomes [18].

Ensuring correct exercise technique is also challenging due to the specificity of muscle activation required to complete some foot exercises [19]. Biofeedback has been used to improve both adherence to regular exercise practice [20] and movement patterns, for gluteus medius activation post stroke [21], muscle re-education to improve gait parameters in older adults with Alzheimer’s [22] and increase abdominal muscle activity in women with chronic low back pain [23]. Using a device that provides real time biofeedback of plantar arch pressure to improve foot muscle control, while recording foot placement and quantity of practice, may improve exercise adherence, performance skill, and foot function.

We designed and constructed a biofeedback device, known as ‘Archercise’, to assist with strength training of foot arch muscles (Patent pending: PCT/AU2016/050437). The Archercise device simultaneously measures and provides real time biofeedback of arch movement and foot location via pressure change in an inflatable arch bladder and sensors embedded in a footplate, respectively. Biofeedback is provided via a computer interface displaying arch movement and foot location. The aim of this study was to investigate the feasibility of using Archercise to provide biofeedback of correct arch movement and foot location during four foot exercises.

## Methods

A cross-sectional observational study design with a single group using repeated measures was conducted. Thirty participants were recruited from the University of Sydney and the general population via advertisement. All data was collected at the Health Sciences laboratory. Invited participants were healthy adults (aged 18 to 68 years) able to walk 50 m barefoot unaided. Study exclusion criteria were: presence of a peripheral or inherited neuropathy (e.g. Diabetes or Charcot-Marie-Tooth disease), an injury affecting foot or lower limb joint motion, history of foot surgery or severe foot pain (≥70 on a 0–100 point scale) in the previous 6 months. The University of Sydney Human Research Ethics Committee approved the study (Project No. 2016/188) and participants provided written informed consent.

### Physical characteristics

All testing for each participant was performed on one occasion. Age, sex, height, weight and BMI were collected. Foot length was measured from the dominant foot (determined by asking with which foot they kicked a ball). Foot alignment was measured using the Foot Posture Index (FPI). The FPI is a reliable weight bearing measure consisting of six items, summed to provide a score from − 12 to + 12 for a supinated or pronated foot, respectively [24].

### Archercise biofeedback device

**Fig. 1:**
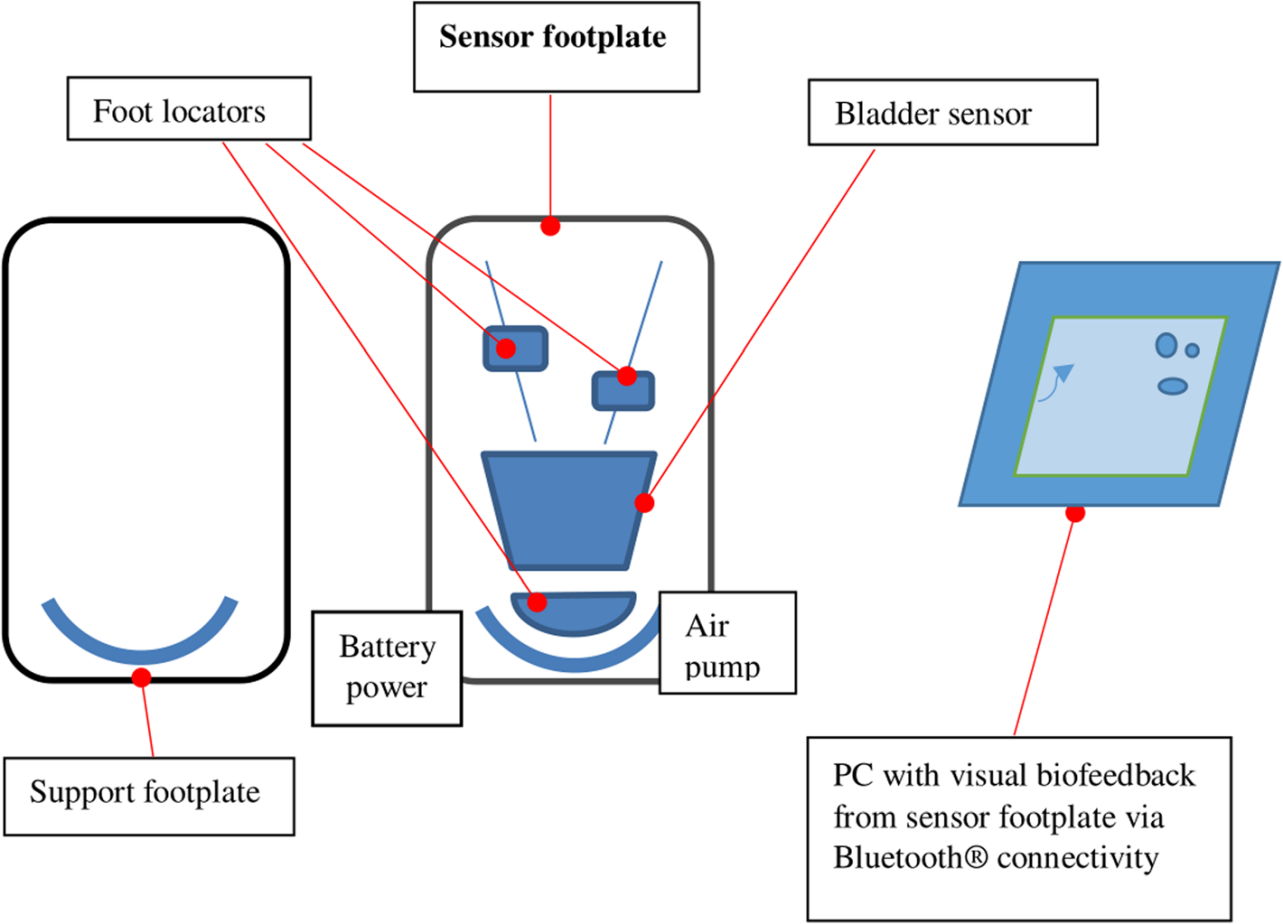
Block diagram of the Archercise device design

Archercise is a non-invasive medical device designed and constructed to assist with exercising foot muscles using the correct technique. Archercise consists of two sensor systems: an electronically controlled inflatable bladder with air pump and air release valve; and adjustable toe and heel sensors embedded into a rigid footplate protected by a removable silicon cover. The device is wirelessly connected to a laptop with a custom designed graphical user interface (“Archercise GUI”), with control buttons to operate all electronic systems in the sensor footplate (Fig. 1). The Archercise GUI provides dual visual feedback via guidance lines and data saving capability. Archercise GUI also calculates a variety of variables generated from the raw data (Additional file 1). The Archercise device measures change in the bladder pressure caused by foot arch movement and foot location using footplate sensors to provide corresponding real-time biofeedback by means of a visual display to actively guide the user’s foot during a series of foot exercise tasks.

### Archercise exercise protocol

**Fig. 2:**
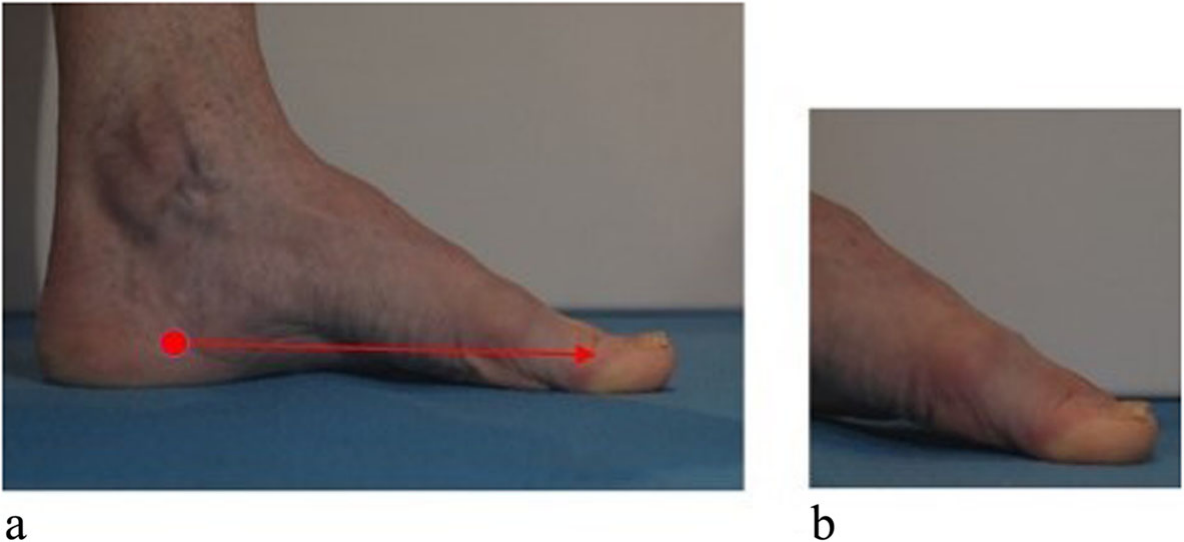
Foot doming (**a**), with detail of arch and metatarsophalangeal joints lifting (**b**)

The components of the Archercise device were explained to the participants, who were then taught a specific arch elevation manoeuvre similar to the short foot exercise [10]. This involved performing an elevation and lowering of the longitudinal plantar arches and metatarsophalangeal joints, pressing elongated distal toes spread out and down [25], while drawing the heel slightly towards the toes (Fig. 2).

### Archercise exercise protocol

**Fig. 3:**
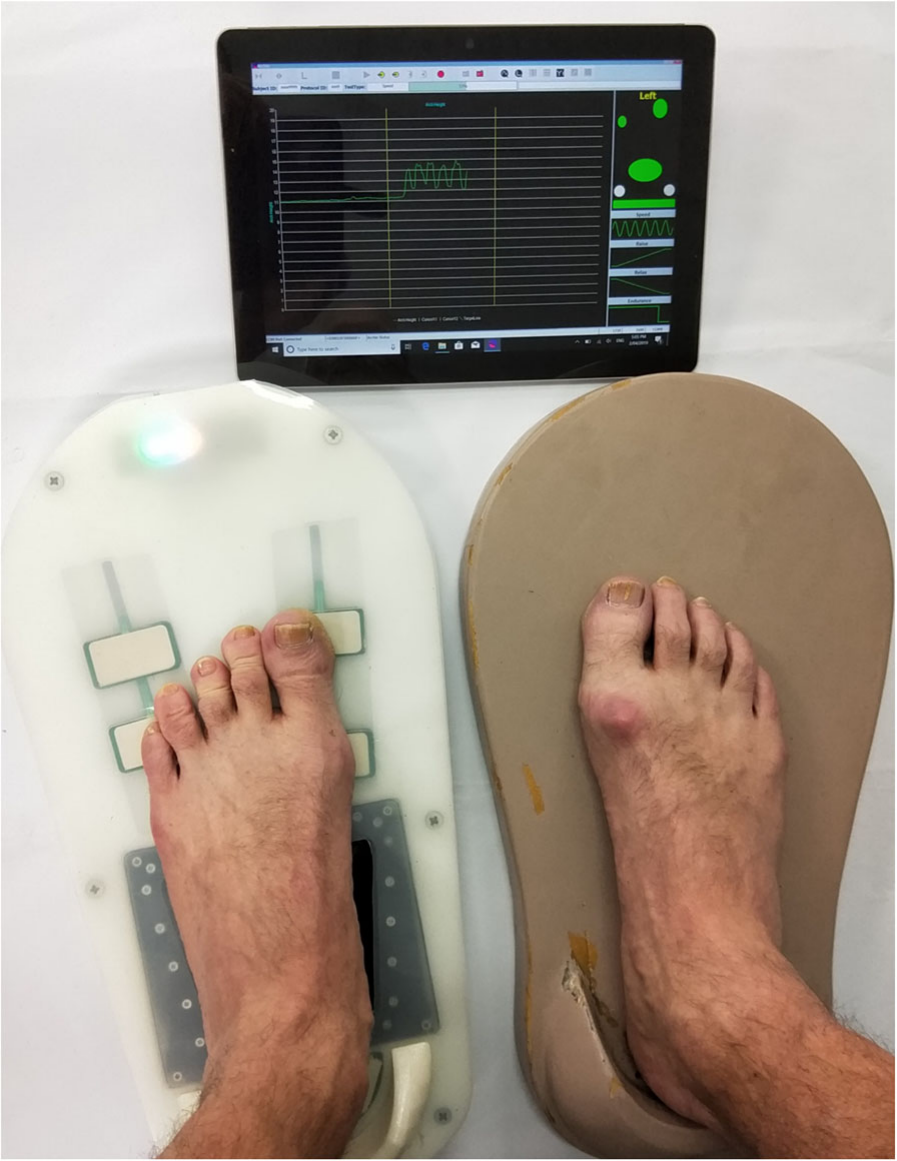
Participant using Archercise device

Participants sat with their knees at about 90°. Before testing, participants rested their dominant foot on the sensor platform to position the foot locator sensors directly under their great and fifth toes. After adjusting the toe sensors, the protective cover was installed, and participants placed their dominant foot on the Archercise device. The bladder was then enabled via an internal self-inflating pump that operates at 50% pressure capacity, thereby adapting to the majority of plantar arch shapes or sizes (Fig. 3).

Participants were familiarised with the specific movement required for each of the four exercises:

1. Speed: sequential isotonic muscle contractions to elevate and lower the arch in as large a range as possible and as quickly as possible. Completed in 10 s during a 30 s data capture mode.
2. Arch elevation: gradual isotonic concentric contraction of the foot arch muscles to lift the arch as high as possible. Completed in 10 s during a 30 s data capture mode.
3. Arch lowering: pre-test arch elevation then a gradual isotonic eccentric contraction of the foot arch muscles, to lower the arch. Completed in 10 s during a 30 s data capture mode.
4. Endurance: elevate the arch and maintain that controlled elevation in an isometric contraction of the foot arch muscles. Completed in 90 s during a 130 s data capture mode.

All data was saved for a minimum of 10 s pre and post exercise performance to ensure clean data capture. To mitigate any early skill learning affect between trials [26], participants practiced to ensure correct execution of the arch elevation and lowering manoeuvres before using the Archerciser device. Three correctly executed repetitions were recorded for each exercise. The set of four exercises were repeated three times on the Archerciser device. The first and second trial of the four foot exercises were completed without biofeedback, with timing for exercise completion provided by an observable stopwatch, to assess consistency of the exercise protocol, then the third trial used the biofeedback interface to assess the effectiveness of the Archerciser biofeedback device.

### Outcome measures

Foot arch pressure was measured with the Archercise device by registering change in pressure in the inflatable bladder under the arch of the foot and simultaneously monitoring foot location using the sensor footplate. Archercise GUI recorded the signals generated by the sensor system and custom software, and determined the following variables:

1. Speed: number of cycles, mean amplitude, amplitude coefficient of variation, mean period, period coefficient of variation.
2. Arch elevation: relative range, slope, coefficient of determination.
3. Arch lowering: relative range, slope, coefficient of determination.
4. Endurance: relative range, mean range, coefficient of variation, area ratio.

Foot location adherence was also calculated for all exercises by determining the percentage of time the great toe, fifth toe and heel correctly contacted the footplate sensors during testing. An additional file provides detailed information on the selection of variables and data calculations (Additional file 1).

Participants were also asked if Archercise with biofeedback helped perform the four foot exercises with correct technique based on a validated patient satisfaction survey [27]. The survey included a ‘yes/no’ response regarding the perceived usefulness of the device, and a series of 0- 100 mm visual analogue scales (with 0 being harder and 100 easier) on perceived ability to complete the exercises with Archercise biofeedback. An option for comments was provided at the end of each survey.

### Statistical analysis

Data were collected and managed using Archercise GUI, custom software and REDCap (Research Electronic Data Capture, Nashville, TN USA) [28]. Statistical analysis was performed using SPSS Windows v22.0 (IBM SPSS Inc., Chicago, IL) for Archercise data and SAS 9.4 statistical package for survey data. Descriptive statistics were generated to characterise the sample. Pearson’s correlation analysis was conducted between the FPI and the Archercise variables. One-way repeated measures ANOVA with pairwise comparisons were computed to assess the consistency of the exercise protocol between trial 1 and trial 2 (prior to biofeedback), and the effectiveness of the Archercise biofeedback device between trial 2 and trial 3. Perceived ability to complete exercises were analysed with paired sample t-tests. Results were considered significant if *p* < 0.05.

## Results

Participant characteristics are described in Table 1. Twenty two (73%) participants exercised or played sport often or always. Six (20%) participants reported previous foot problems in the last 6 months: in the heel (*n* = 1), great toe with mild hallux valgus (*n* = 3), and swelling in the lesser toes, or cramps in the fore and mid foot (*n* = 2). Of those with foot problems, one reported no pain and five (17%) reported occasional foot pain, 2 reported pain in the non-dominant foot, 3 reported pain ranging from 8 to 30 mm on a 0–100 mm scale in the dominant foot. Significant correlations were found between the dominant FPI with the speed cycle (*p* = 0.015, r = 0.404) and endurance area ratio in bout one (*p* = 0.027, r = 0.404), and with foot location during the endurance exercise (*p* = 0.047, r = − 0.366) in bout three.

**Table 1:** Participant characteristics of the sample (*n* = 30)

Participant characteristics	Value
Age, y	37.47 ± 12.7
Sex, Female no. (%)	19 (63%)
Body weight, kg	67.6 ± 15.3
Height, cm	167.2 ± 7.2
BMI, kg/m^2^	24.0 ± 4.2
Dominant foot, right	28 (93%)
Foot Posture Index (score)	0.8 ± 1.1
Foot length, cm	24.6 ± 1.5

Values are mean ± SD unless otherwise stated Abbreviations: *y* Year, *kg* Kilogram, *m* Metres, *BMI* Body mass index, *cm* Centimetres

Regarding consistency of the foot exercise protocol, there was no difference between trial 1 and 2 for 17 of 19 (89%) arch movement or foot location variables using Archercise (*p* > 0.05). Speed foot location (*p* = 0.021) and arch lowering slope (*p* = 0.026) worsened during trial 2 (Table 2). Regarding effectiveness of Archercise with biofeedback, foot location adherence improved for all exercises (*p* = 0.003–0.008), as well as coefficient of determination for controlled arch elevation (*p* < 0.0001) and endurance area ratio (*p* = 0.001). Variables that worsened with biofeedback were arch elevation relative range (*p* = 0.004) and slope (*p* = 0.028), and endurance relative range (*p* < 0.001) (Table 2). Effect size (partial eta squared) with a significant positive effect for biofeedback on foot location: speed partial η^2^ = 0.359, concentric elevation partial η^2^ = 0.273, eccentric lowering partial η^2^ = 0.317 and endurance partial η^2^ = 0.466; coefficient of determination for controlled arch elevation partial η^2^ = 0.609 and endurance area ratio partial η^2^ = 0.507. Variables with a negative effect for biofeedback were arch elevation relative range partial η^2^ = 0.382 and slope partial η^2^ = 0.217, and endurance relative range partial η^2^ = 0.615.

**Table 2:** Trial 1 and 2 shows the consistency of the foot exercise protocol without biofeedback, and Trial 3 shows the effectiveness of the Archercise device with the biofeedback

Variable	Trial 1	Trial 2	Trial 3
	Mean ± SD	Mean ± SD	Mean ± SD
Speed cycle	13.0 ± 5.0	13.6 ± 5.6	12.9 ± 5.9
Speed mean Amplitude	2.6 ± 1.4	2.7 ± 1.2	2.9 ± 0.9
Speed Amplitude CoV	2.8 ± 1.9	2.9 ± 2.7	2.1 ± 1.5
Speed mean Period	0.8 ± 0.3	0.8 ± 0.3	0.8 ± 0.3
Speed Period CoV	0.5 ± 0.5	0.4 ± 0.7	0.5 ± 0.7
Speed foot locator compliance	36.6 ± 34.4	21.6 ± 25.4*	30.0 ± 31.6^#^
Arch elevation relative range	4.0 ± 1.8	4.3 ± 1.5	3.3 ± 0.8^#^
Arch elevation slope	0.4 ± 0.2	0.4 ± 0.2	0.3 ± 0.1^#^
Arch elevation CoD	0.7 ± 0.3	0.7 ± 0.2	0.9 ± 0.1^#^
Arch elevation foot locator compliance	37.9 ± 37.5	32.6 ± 33.6	52.0 ± 34.1^#^
Arch lower relative range	4.2 ± 1.7	3.8 ± 1.7	3.3 ± 0.9
Arch lower slope	− 0.4 ± 0.2	−0.35 ± 0.2*	−0.3 ± 0.1
Arch lower CoD	0.9 ± 0.1	0.8 ± 0.2	0.9 ± 0.1
Arch lower foot locator compliance	28.6 ± 29.6	29.5 ± 28.4	50.0 ± 34.2^#^
Endurance relative range	4.5 ± 1.6	4.75 ± 1.7	3.5 ± 1.1^#^
Endurance mean range	3.5 ± 1.8	3.9 ± 1.9	3.4 ± 1.2
Endurance CoV	0.3 ± 0.4	0.1 ± 0.2	0.1 ± 0.1
Endurance area ratio	73.6 ± 22.1	77.3 ± 17.6	89.1 ± 14.1^#^
Endurance foot locator compliance	36.0 ± 35.1	46.0 ± 35.6	70.8 ± 36.4^#^

*Significant difference between trial 1 and 2 (*p* < 0.05). ^#^ Significant difference between trial 2 and 3 (*p* < 0.05)

Legend: CoV coefficient of variation, CoD coefficient of determination. Foot locator variable range from 0 to 100, all other variables range 0–20 arbitrary units

Self-report perception of ability to perform all exercises improved with biofeedback: speed 72.6 ± 14.8 to 81.7 ± 12.6 (*p* = 0.001), arch elevation 71.3 ± 15.1 to 79.9 ± 14.4 (*p* = 0.010), arch lowering 60.6 ± 22.2 to 72.2 ± 18.5 (*p* = 0.020), endurance of maintaining arch elevation 76.2 to 84.5 ± 10.0 (*p* = 0.010). Twenty-nine of 30 (97%) participants reported that Archercise with biofeedback helped correctly perform the exercises. Survey comments are reported in Table 3. There were no adverse events reported.

**Table 3:** Survey comments on Archercise exercise protocol performed with biofeedback

Thematic grouping	Comments
General positive	Everything worked well
	Much easier
Biofeedback helped	Biofeedback made the task easier and my foot muscles seemed to improve
	Everything worked well
	Simply, the visualisation works very well
	The feedback all worked to get the exercise correctly
	The biofeedback was fantastic and was very helpful in the tasks
	Good to get feedback, hard to reach some of the highest markers, helps to recreate the expected patterns
Specific biofeedback GUI	I felt that the graphs were a huge help for me to perform the tasks as they were described to me.
	Seeing my progress on the screen was good
	Seeing the monitor gave good feedback on whether I’m doing the task correctly or not
	The feedback helped to understand the concept of slowly lifting up and lowering down, and having the feedback helped with endurance and know that ‘i’m doing the right thing.
Negative comments	Concentrating on the new task (lowering the arch) and looking at the screen was difficult.
	Fatigue affected the latter results.
**Specific biofeedback components**	
Bladder	Bladder provided useful tactile feedback
	Feeling the bladder under the arch helped enormously with arch awareness.
	The pressure under the arch was very helpful and informative
Foot locators	Works well to see how your foot position changes are shown on the screen, to feedback if you are doing it correctly, especially with the speed test.
	…. assisted specially with the little toe
Visual display	It was easier to understand what I was supposed to do with visual feedback.
	The biofeedback made a huge difference in how I perceived that I performed the tasks, especially the arch relaxing task.
	The screen really helps to complete the tasks.
	The screen was a good stimulus to do the exercises properly
	Pressure waves & guidance line of best fit (helped).
	The guide and feedback when doing lifting up and lowering down helped with controlling the movement.
	Help in most tasks but not helpful for me with the eccentric task^a^.

Legend: eccentric task^a^, arch lowering

## Discussion

The main finding of this study was that the Archercise biofeedback device helped almost all participants perform the four foot exercises correctly. Biofeedback from the footplate sensors improved foot placement during all exercise tasks, demonstrating that speed of arch elevation and lowering, controlled arch elevation and arch lowering, and endurance of maintaining an elevated arch were performed with the correct technique once the biofeedback device was activated. Archercise also demonstrated that the exercise protocol displayed good consistency, with 89% of variables unchanged between trials, prior to activating the biofeedback device.

Perceived ability to complete the arch lowering exercise was the most difficult (60.6 ± 22.2) and the endurance of maintaining an elevated arch was the easiest to perform (76 ± 16.2) on the 0–100 mm scale. With biofeedback, speed of arch elevation and lowering improved by 12.5%, controlled arch elevation by 12%, arch lowering improved by 19%, and endurance of maintaining an elevated arch improved by 10.8%. Interestingly, even though eccentric training was considered more challenging [29] participant perceived ability to complete the arch lowering exercise was most improved with biofeedback. Although the clinical meaningfulness of the magnitude of the differences is yet to be determined, the considerable perceived improvement in the arch lowering exercise and comments such as: “*the biofeedback made a huge difference in how I perceived that I performed the tasks*, *especially the arch relaxing (lowering) task*” (Table 3) substantiates the positive effect biofeedback had on perceived performance skill even for the most difficult exercise. Since self-efficacy is inherently motivating [30], this positive reinforcement may also improve exercise adherence.

The Archercise biofeedback device corrected foot location motor performance skill for all four exercises. This occurred even for the speed task, with its complex requirements to complete a series of sequential isotonic contractions, to elevate and lower the arch as quickly as possible. The substantial improvement for the foot location task emphasises the importance of feedback to enhance correct foot placement, which may assist effective intrinsic foot muscle recruitment. Since biofeedback was always undertaken in bout three, the improvement in performance may be due in part to motor learning. Even so the strength of the association for improving foot location when using biofeedback was large for all four exercises [31]. Also as early skill development has been reported to be quite rapid [32], due to the practice requirements performed before data collection, we considered that there was likely to be minimal change to the early learning effect.

Relationships between Archercise variables and the dominant FPI were explored. Only three weak associations were found between the variables, with inconsistency across the bouts. The weakness of the correlations could be due to small numbers and the limited variation in the FPI of participants. Two of the Archercise variables with significant associations to the FPI, are based on the ability to maintain foot position while performing the endurance exercise. Interestingly one has a positive and one a negative correlation, with no consistency across bouts. Diverse results have been also reported on the relationship between the FPI and dynamic foot function in some studies [33, 34]. As foot motion is complex and varies from person to person [35], further research comparing other measures of foot function such as motion capture data rather than the FPI may validate the Archercise’s measures of foot motion and motor skill.

A significant decrease in arch relative range was observed in arch elevation and endurance tasks, and arch elevation slope also worsened with biofeedback. Muscle fatigue may have contributed to reduced arch relative range. A significant decrease in standing navicular height has been reported post a fatigue task (*p* < 0.0005) [36]. However, the study required several sets of 75 repetitions of isotonic flexion contractions of the intrinsic foot muscles against a 4.55 kg weight to observe a fatigue effect [36]. Suggesting abductor hallucis, which has a prime role in maintaining the height of the medial longitudinal arch [37] is considerably resistant to fatigue [38]. The reduction in arch elevation range and, therefore reduced slope (angle of elevation) were most likely due to participants conscious attention to improving muscle control [39] and prioritising skill acquisition [40]. The use of a guidance line appeared to encourage participants to limit range in preference for control of arch movement with comments such as the guidance line “*made it harder to reach highest marker, while helps to recreate expected pattern*”. Similarly, the significant improvement in keeping to the line of best fit for arch elevation and the ability to maintain initial range for the duration of the endurance exercise using biofeedback, showed the participant’s ability to improve performance skill using guidance lines [41].

Biofeedback has been used with good effect in rehabilitation programs [42], with evidence showing it provides multiple benefits post stroke, with: improved walking ability [43], increased gait symmetry, and loading on the affected side [21]. A Systematic review of gait retraining with biofeedback found the initial treatment effect varied from moderate to large, however as none of the included studies provided follow up, it was not known if any treatment effect was maintained [44]. Even so, home exercise programs that included biofeedback have been shown to improve a range of diverse problems such as gait insufficiency post incomplete spinal cord injuries [21], and sphincter control for fecal incontinence [45]. Complex biofeedback systems for neuromotor rehabilitation intended for home use have been developed, such as wearable technology [46] and game based exercise [47]. While using visual biofeedback has been reported to add effectiveness during training for improved dynamic balance [47], weight shifting in standing, and reduced postural sway [48], a drawback is, these systems do not provide a direct measure of any given exercise. Archercise provides biofeedback via a unique real time measure of change in arch height and foot location to specifically aid strength training of foot muscles.

Aside from the positive self-reported effects of the Archercise biofeedback device, participants also specifically commented on the sensation of the inflatable bladder under their arch, such as: “*Feeling the bladder under the arch helped enormously*”. It is likely the bladder under the arch may offer improved plantar sensory input. In addition, participants reported that the foot location sensors “*Works well to see how your foot position changes are shown on the screen, to feedback if you are doing it correctly, especially with the speed test*” and the real-time display provided a “*screen with good stimuli to do the exercises properly*”. The visual depiction of pressure under the arch simultaneously with foot location, provided self-supervision of correct form thus reducing the likelihood of substitution movements, such as foot supination instead of arch elevation.

### Study limitations

This study has several limitations. First, only 30 primarily active adults participated in this study and the results should not be generalised outside this population. Further studies with patient groups known to have foot muscle weakness such as those with peripheral neuropathy or an older population are necessary to determine the effectiveness of the biofeedback based foot exercise program. Second, as there was no control group, the final outcomes may be biased. Third, there was no blinding of either participant or assessor, which could be a source of further bias. Fourth, the choice or sequence of exercise tasks or fatigue may have also affected the results. Therefore, longitudinal studies trialling different exercise sequences with participants randomly selected into active intervention or control groups, with assessor blinding would provide verification on the usefulness of the intervention. In addition, it is also recommended that the outcome variables (Additional file 1) be examined with different doses of exercise and diverse patient groups, to determine exercise specificity and responsiveness. Only one single manoeuvre “arch doming” was tested. Arch doming was performed in four different ways to ensure a range of muscle abilities related to functional performance were tested. The speed exercise was included as rapid rhythmic movement improves toe flexion strength [49–51] and has a positive effect on gait and improves reaction time to perturbations in older people [52]. Toe flexion strength training improves standing long jump performance [50] and jumping distance [53]. Traditionally strength training has focused on concentric exercise particularly if muscle hypertrophy is the goal [54]. Increased fibre type 11 [55], fibre length and distal hypertrophy occurs more with eccentric muscle training than with concentric training [56]. Since strength training requires both concentric and eccentric exercise and hallux plantar flexion strength is reported as one of the most consistent significant and independent predictors of balance and functional test performance in older adults [57], concentric and eccentric exercises were performed separately. The endurance exercise task was included as the ability to maintain arch height, may also assist help maintain balance [58]. Additional exercises could also be explored in order to generalise the benefits of Archercise to other resistance strength training programs. This study only used the seated position, future studies progressing to double and single leg stances or with an incline would enable more challenging training programs to be trialled.

## Conclusion

In conclusion, the Archercise biofeedback device appears to be a safe and feasible system to assist healthy participants without foot pathologies perform foot doming exercises and improve adherence to the correct technique. The Archercise device merits further research to explore the longitudinal benefits in community-based clinical trials to treat foot muscle weakness.

## Supplementary information

**Additional file 1.** Archercise data outcome variables; description, calculation and performance indicator.

## Data Availability

The data sets used and/or analysed during the current study are available from the corresponding author on reasonable request.

## Abbreviation

GUI: Graphic user interface
