# Supplementary material for: Feasibility of the Archercise biofeedback device to strengthen foot musculature
Source: J Foot Ankle Res. 2020 Jul 13;13:43. doi: 10.1186/s13047-020-00394-z (PMC7359285; doi:10.1186/s13047-020-00394-z)
Supplement: Supplementary file 1 — Additional file 1. Archercise data outcome variables; description, calculation and performance indicator. [file 13047_2020_394_MOESM1_ESM.docx]

**Supplementary Table S1:** Archercise data outcome variables; description, calculation and performance indicator

| **Exercise** | **Variable** | **Description and Calculation** | **Performance indicator** |
| --- | --- | --- | --- |
| Speed | | Uses a peak detection algorithm to identify each peak and trough of a sinusoidal waveform. |  |
|  | Cycles | Number of peaks following the first peak in an interval. Peaks between X1 and X2 in 10 seconds. | How quickly the participant can sequentially elevate and lower their arch. |
|  | Mean amplitude | Mean magnitude of the wave or the difference between a peak and trough. | Range for the arch lifting and lowering during the fast muscle activation and relaxing.* |
|  | Amplitude coefficient of variation | Variation of the amplitude normalised to the mean.  (CoV = Standard Deviation/Mean). | Quality of the arch lift and lower. A decrease in the CoV denotes higher performance skill. |
|  | Mean period | Mean period (time interval) between each peak. | How quickly the participant can activate and relax the small foot muscles under the arch. |
|  | Period coefficient of variation | Variation of the period normalised to the mean  (CoV = Standard Deviation/Mean). | A decrease in the CoV denotes higher performance skill. |
| Elevation and lowering | |  |  |
|  | Relative range | Difference between the maximum and minimum arch height, defined by the vertical cursors X1 to X2. | How much the participant can lift or lower the arch from relaxed to as high as possible or from as elevated as possible to relax the arch.* |
|  | Slope | Ratio of vertical change over horizontal change or time interval (Slope = Range(X1-X2) / Interval) in 10 seconds. | How well the participant can control the gradual concentric or eccentric contraction of the small foot muscles. |
|  | Coefficient of determination | r^2^ is a statistical measure of how well a regression line approximates the real data points collected, calculated by (R^2^ = 1 –SSres/SStot) ,while following a guidance line calculated by using a pre test practice (slope = Range (X2 – X1) / Interval). | An increase in the coefficient of determination (closer to 1) denotes higher performance skill of the particpants ability to follow the guidance line. |
| Endurance | |  |  |
|  | Relative range | Range of arch elevation from pre elevation to maximum arch elevation. | How much the participant can elevate the arch.* |
|  | Mean range | Mean range of arch elevation over the time interval of the exercise. | Determines the participants ability to maintain arch elevation over the duration of the exercise. |
|  | Coefficient of variation | Variation or the consistency of the participants arch elevation over 60 seconds  (CoV = Standard Deviation/Mean). | Ability to maintain a steady small foot muscle contraction or arch lift for 60 seconds. A decrease in the CoV denotes higher performance skill. |
|  | Area ratio | Area under the pressure signal divided by the total possible area expressed as a percentage (total possible area is 80% of the user’s initial range multiplied by test duration). | Increased area ratio indicates improved performance skill by consistently maintaining an increased arch elevation. |
| All exercises | |  |  |
|  | Foot location | Percentage of time the great toe, fifth toe and heel contacted the membrane switches during testing. | Increased foot location indicates improved ability of participant to maintain correct contact with heel, great and fifth toe membrane switches, ensuring foot alignment is kept within the same plane during each exercise. |

Key: * Comparisons can be made with mean amplitude or relative range to explore any relationship between range and exercise: X1, first cursor; X2, second cursor; CoV, coefficient of variation; CoD coefficent of determination; SSres = sum of residuals = ∑(yi – Yi)^2^ from i = 1 to n; SStot = total sum of squares = ∑(yi – meanY)^2^ from i = 1 to n
